# Incidence of death and its predictors among neonates admitted with sepsis in referral hospitals, northwest Ethiopia, a prospective cohort study

**DOI:** 10.3389/fped.2023.1129924

**Published:** 2023-04-13

**Authors:** Saron Abeje Abiy, Yaregal Animut, Worku Mequannt Ambaw, Getie Mihret Aragaw, Bayew Kelkay Rade

**Affiliations:** ^1^ Department of Clinical Midwifery, School of Midwifery, College of Medicine and Health Sciences, University of Gondar, Gondar, Ethiopia; ^2^ Department of Epidemiology and Biostatistics, Institute of Public Health, College of Medicine and Health Sciences, University of Gondar, Gondar, Ethiopia; ^3^ Department of General Midwifery, School of Midwifery, College of Medicine and Health Sciences, University of Gondar, Gondar, Ethiopia

**Keywords:** death, incidence, neonates, predictors, sepsis, Ethiopia

## Abstract

**Background:**

Each year, approximately 2.7 million neonates die in their first month of life worldwide, and the majority of these deaths occur in low-income countries. According to the Global Burden of Disease estimation, 1.3 million annual incident cases of neonatal sepsis were reported worldwide, resulting in 203,000 sepsis-attributable deaths. Little is known about the time to death of neonates and predictors after admission with a diagnosis of sepsis. This study aimed to assess the incidence and predictors of death among neonates admitted to the neonatal intensive care unit with a diagnosis of sepsis in referral hospitals in Northwest Ethiopia.

**Methods:**

A multicenter prospective follow-up study was conducted from November 11 to December 7, 2021. A stratified random sampling technique was employed to select 412 neonates. Neonates admitted with sepsis were followed until they develop event for a maximum of 28 days of age. A face-to-face interview was conducted with the mother of the neonate using a pretested and structured questionnaire, and neonatal charts were reviewed to collect baseline factors. Data were entered into Epi-data version 4.6 and exported to STATA version 14 for analysis. A bivariable and multivariable exponential Cox regression model was fitted to identify predictors of death. The adjusted hazard ratio (AHR) with 95% CI was calculated, and statistical significance was declared at a *P*-value of 0.05 in the multivariable analysis.

**Results:**

A total of 75 (18.47%) neonates died during the study period, with a 95% CI of 14.82–22.60. The incidence rate of death was 28 (95% CI, 22, 35) per 1,000 person-days of observation, with a total follow-up time of 2,677 person-days of observation. Birth weight (<2,500 g) (AHR = 2.12, 95% CI: 1.01, 4.43), prematurity (AHR = 2.06, 95% CI: 1.02, 4.15), duration of labor >24 h (AHR = 3.89, 95% CI: 1.38, 11.01), breast feeding (AHR = 0.43, 95% CI: 0.23, 0.80), having respiratory distress syndrome (AHR = 1.77, 95% CI: 1.02, 306), oxygen saturation less than 90% (AHR = 2.23, 95% CI: 1.02, 306) were significant predictors of death among neonates admitted with sepsis.

**Conclusion and recommendation:**

The incidence of neonatal mortality in this study was high. Early detection and appropriate management of patients’ presentations like respiratory distress syndrome and low oxygen saturation are necessary to reduce neonatal sepsis-related mortality. Special attention should be given to low birth weight and premature neonates and mothers should be encouraged to breastfeed their newborns after delivery.

## Introduction

Neonatal sepsis is a systemic infection that occurs within the first 28 days of a newborn's life and includes septicemia, pneumonia, meningitis, arthritis, osteomyelitis, and a urinary tract infection (1). The 2005 International Pediatric Sepsis Consensus Conference defined neonatal sepsis as a systemic inflammatory response syndrome as a result of suspected or proven infection in a neonate (2). Based on the onset of symptoms, neonatal sepsis could be either early-onset sepsis (EOS) or late-onset sepsis (LOS). EOS manifests clinically within the first 72 h and is transmitted both prenatally and intrapartum. LOS, on the other hand, manifests clinically after 72 h of childbirth and is primarily acquired horizontally from the environment (1). Neonatal sepsis ranges from subclinical infection to severe focal or systemic manifestations like; respiratory distress, jaundice, vomiting, and poor feeding (2, 3).

Neonates are predisposed to infections during the perinatal period due to multiple exposures and a relatively compromised immune system. Neonatal conditions like low birth weight, an Apgar score of 7 or less at 5 min, not crying immediately at birth, and gestational age of 37 weeks or less were found to be risk factors for neonatal sepsis (4, 5). Previous pieces of evidence also showed that maternal age, wealth status, maternal urinary tract infections or sexually transmitted infections, intrapartum fever, antenatal care, the starting time of breastfeeding, resuscitation at birth, and nasogastric tube insertion are associated with neonatal sepsis (3, 4).

Neonatal mortality is a major public health problem worldwide, particularly in low-income countries. Every day, approximately 7,000 neonates die in their first month of life worldwide, and the majority of these deaths occurred in low-income countries, specifically in Sub-Saharan Africa, where there is a high burden of neonatal mortality which accounts for an estimated 49.6% of all under-five deaths. According to the Global Burden of Disease (GBD) Study in 2016–2017, an estimated 1.3 million annual incident cases of neonatal sepsis were reported worldwide, resulting in 203,000 sepsis-attributable deaths from which around 75% of the burden occurs in developing countries (6, 7). In Ethiopia, neonatal mortality is unacceptably high at 30 per 1,000 live births, which is far from the global Sustainable Development Goal (SDG) target of reducing neonatal mortality to 12 per 1,000 live births by 2030 (8) and one major cause of neonatal mortality in the country is neonatal sepsis (9). Even though there are effective treatments for neonatal sepsis, a significant number of neonatal deaths have been reported in different studies among hospital-admitted neonates (1, 10–12). There were study gaps in determining predictors for sepsis-related mortality in neonatal intensive care units in our country and the study area in particular. As a result, this study aimed to determine the incidence of death and predictors among neonates admitted with sepsis and the findings will help policy makers and clinicians in designing strategies to improve the quality of neonatal health services in the study area and at the country level, and thus leads to decreasing neonatal morbidity and mortality.

## Methods and materials

### Study design and period

A multicenter prospective follow-up study design was conducted from November 11 to December 7, 2021.

### Study area and setting

This study was conducted in the neonatal intensive care unit (NICU) of five referral hospitals in northwest Ethiopia. We have included all 5 referral hospitals in Northwest Ethiopia namely; the University of Gondar Comprehensive Specialized Hospital, Debre Tabor Comprehensive Specialized Hospital, Debre Markos Comprehensive Specialized Hospital, Felege Hiwot Comprehensive Specialized Hospital, and Tibebe Ghion Specialized Hospital (13).

### Population and sample

All neonates with a diagnosis of neonatal sepsis admitted to the Neonatal Intensive Care Unit (NICU) in referral hospitals of North West Ethiopia during the study period were the study population. All neonates with neonatal sepsis admitted to neonatal intensive care units and start treatment were included in this study and neonates with major congenital abnormalities were excluded from the study. The sample size of the study was calculated using the following assumptions; the probability of an event from the previous study (14.7%), the incidence of mortality among admitted neonates with sepsis from a study conducted at Mizan Tepi University Teaching Hospital, Southwest Ethiopia (14). Probability of type 1 error or alpha 0.05, power of the study 80%, 95% confidence level, and 10% withdrawal probability from the study. The sample size was also calculated considering different hazard ratios (HR) of significant predictors from the previous studies including birth weight neonatal age at admission, maternal infection, and length of hospital stay and the largest sample size was considered as a final sample size.

$$E=\frac{4{\left(Z\alpha //2+Z\beta \right)}^{2}}{{\left(\ln HR\right)}^{2}}$$

$$E=\frac{4{\left(1.96+0.84\right)}^{2}}{{\left(\ln2.13\right)}^{2}}$$

E=55

Where *E* is the number of required event for the study.

Then the required total number of individuals was found from the formula:

$$N=\frac{E}{P(E)}$$

$$N=\frac{55}{0.147}$$

N=374

Where, *N* is calculated sample size and *P*(*E*) is the probability of an event from the previous study. Finally considering a 10% withdrawal probability, we use a final sample size of 412.

A stratified random sampling was employed to select neonates admitted with sepsis in the selected referral hospitals. The sample size was allocated proportionally to each hospital based on the hospital’s patient flow (average number of neonates admitted in each hospital). Then study subjects were selected using systematic random sampling techniques in which every other neonate admitted with sepsis were included in the study during the study period.

### Variables of the study

#### Dependent variable

Time to death of neonates admitted with sepsis.

#### Independent variables

**Socio-demographic factors of the mother** (age, sex, residence, income, educational level).

**Obstetric factors** (gravidity, mode of delivery, duration of delivery, history of ANC, place of delivery, Premature rupture of membranes, abortion, stillbirth).

**Clinical characteristics of neonate** (gestational age, birth weight, dehydration, breastfeeding status, oxygen saturation, jaundice, RDS, asphyxia, seizure, hypoglycemia).

### Operational definition

**Time to death**; is calculated from the time of the initiation of treatment till the death of the neonate.

**Censored**: neonates who did not experience death during the follow-up period including lost follow-up, referred to other health institution, discharged with improvement, or stayed with admission beyond 28 days of neonatal age.

**Neonate**: newborn from birth to 28 days old (15).

**Neonatal sepsis**: sepsis diagnosed clinically by professionals or attending physicians during admission of the neonate (16).

L**ow birth weight**: a birth weight of less than 2,500 g at birth (17).

### Data collection tools and procedure

A pretested and structured Amharic-version questionnaire was used to collect the data. Maternal sociodemographic characteristics were collected using face-to-face interviews, and obstetrics and baseline clinical characteristics of the neonate were recorded during admission. Then the neonates were followed until discharged, died, and were lost to follow-up. One MSc nurse supervisor and two BSc data collectors were recruited in each referral hospital to collect the data.

The data collectors and supervisors were trained for 1 day on the objective of the study, how to extract the treatment outcome from a chart, and the basics of interviewing before the actual data collection. A structured questionnaire was first prepared in English and then translated to Amharic and back to English by language experts to check consistency and conceptual similarity. The data collection process was closely monitored by the principal investigator and the supervisors throughout the data collection period. Filled questionnaires were checked regularly for completeness of information, and any problems were immediately discussed with the data collectors. A pre-test was conducted on 5% of the sample size in Debre Markos Referral Hospital before the actual data collection period.

### Data processing and analysis

Data were entered into EpiData version 4.6.0.6 and exported into STATA version 14 for analysis. Frequency tables, percentages, and median were used to describe the data. The hazard of death among neonates admitted with sepsis across different categories of the covariates was compared by using the nelson Aalen curves and log-rank tests. The Cox proportional hazard (PH) assumption was checked graphically by the log-minus-log function and statistically by Schoenfeld residual (global) test and the assumption was fulfilled. Cox PH and three parametric models (Exponential, Weibull, and Gompertz) were fitted to identify the predictors of death among neonates admitted with sepsis. The best model was selected using Akaike Information criteria (AIC), Bayesian information criteria (BIC), and log-likelihood criteria. The exponential regression model was selected as the best model with the lowest AIC and BIC. The Goodness of fit of the model was assessed by using the cox-snell residual technique. The Shared Frailty model was checked to see a random effect, an unobserved multiplicative effect on the hazard rate, for all individuals in the same hospital. But the frailty for the hospital was not statistically significant (theta = 0.24, *x*^2^ = 0.11, *P*-value = 0.372).

A bivariable and multivariable exponential regression model was fitted to identify predictors of mortality. Variables with a *P*-value of 0.2 in the bivariable analysis were entered into the multivariable analysis. The adjusted hazard ratio (AHR) with 95% CI was calculated, and statistical significance was declared at *P*-value of 0.05 in the multivariable analysis.

## Results

### Socio-demographic characteristics of mothers

A total of 406 neonates were included in the study, providing a response rate of 95.5%. The mean age of the mothers was 29 years with a standard deviation of 4.7 years. More than two-thirds 276 (67.98%) of the mothers of the neonates were between the ages of 25 and 34 years; 384 (94.58%) were married; 123 (30.30%) were unable to read and write, and 198 (48.77%) were housewives (Table 1).

**Table 1:** Maternal socio-demographic characteristics of neonates admitted with sepsis at NICU in referral hospitals of north west Ethiopia, 2021 (*N* = 406).

Variable	Frequency	Percentage (%)
**Age**		
18–24	66	16.26
25–34	276	67.98
≥35	64	15.76
**Educational level**		
Unable to read and write	123	30.30
Read and write	71	17.49
Primary school	52	12.81
Secondary school	82	20.20
College and above	78	19.21
**Religion**		
Orthodox	382	94.09
Muslim	24	5.91
**Residence**		
Urban	225	55.42
Rural	181	44.58
**Marita status**		
Unmarried	14	3.45
Married	384	94.58
Divorced	4	0.99
Widowed	4	0.99
**Occupation**		
Civil servant	63	15.52
Merchant	128	31.53
NGO worker	17	4.19
Housewife	198	48.77
**Family size**		
<5	263	64.78
≥5	143	35.22
**Income**		
<2,000 ETB	62	15.27
2,000-3,499 ETB	136	33.50
3,500-6,999 ETB	95	23.40
≥7,000 ETB	113	27.83

### Baseline clinical characteristics of neonates

Out of 406 neonates enrolled, 234 (57.64%) were males, and the majority of the neonates, 392 (96.55%), were less than 1 week old at admission. About 217 (43.35%) neonates had a birth weight of less than 2,500 g. More than half of the neonates, 230 (56.65%), were initiated breastfeeding, and about 91 (22.41%) of the neonates had RDS and 13 (3.20%) had PNA at admission. Twenty-five (6.16%) of the neonates had a history of previous admission, and more than half (63.55% and 60.34%) had a normal WBC count and platelet count, respectively. Almost all (99%) of the neonates initiated their treatments with ampicillin and gentamicin. The first antibiotic was changed for 57 (14.04%) of the neonates, and 48 (84.21%) of them were due to a poor response to the first antibiotic (Table 2).

**Table 2:** Baseline characteristics of the neonates admitted with sepsis at NICU in referral hospitals of northwest Ethiopia, 2021 (*N* = 406).

Variable	Frequency	Percent
**Sex**		
Male	234	57.64
Female	172	42.36
**Age**		
≤7 days	392	96.55
8–28 days	14	3.45
**Birth weight**		
Low	217	53.45
Normal	178	43.84
Macrosomia	11	2.71
**Pulse rate (beat/minute)**		
<100	12	2.96
100–160	332	81.77
>160	62	15.27
**Oxygen saturation**		
<90%	246	60.59
≥90%	160	39.41
**Twin**		
Yes	58	14.29
No	348	85.71
**Breastfeed**		
Yes	230	56.65
No	176	43.35
**RDS**		
Yes	93	22.91
No	313	77.09
**Perinatal asphyxia**		
Yes	13	3.20
No	393	96.80
**Dehydration**		
Yes	48	11.82
No	358	88.18
**Jaundice**		
Yes	90	22.17
No	316	77.83
**Central cyanosis**		
Yes	90	22.17
No	316	77.83
**Bulging fontanel**		
Yes	37	9.11
No	369	90.89
**MAS**		
Yes	30	7.39
No	376	92.61
**Seizure**		
Yes	31	7.64
No	375	92.36
**History of previous admission**		
Yes	25	6.16
No	381	93.84
**Any surgical procedure**		
Yes	3	0.74
No	403	99.26
**WBC count**		
<5,000	17	4.19
5,000–20,000	258	63.55
>20,000	131	32.27
**Platelet count (cell/mm^3^)**		
<150 **× **10^3^	155	38.18
150–450 × 10^3^	251	61.82
**Hypoglycemia**		
Yes	63	15.52
No	343	84.48
**Changed their initial antibiotics**		
Yes	57	14.04
No	347	84.96

RDS, respiratory distress syndrome; MAS, meconium aspiration syndrome.

### Obstetrical characteristics

More than half of the mothers (54.93%) received antenatal care. Thirty-five (8.62%) of the mothers had a history of stillbirth, and 47 (11.58%) had a history of abortion. Sixty-five (16.01%) of the mothers had hypertensive disorders of pregnancy during the current pregnancy; of them, 61 (93.8%) had pre-eclampsia and four (6.22%) had eclampsia. More than three-fourths (82.02%) of the neonates were delivered at hospitals, and more than half (66.75%) were delivered spontaneously. Nearly half (47.04%) of the women had a history of premature rupture of membrane (PROM), of them 16 (8.38%) had a latency period of eighteen and more hours (Table 3).

**Table 3:** Obstetrical characteristics of mothers of the neonates admitted with sepsis at NICU in referral hospitals of northwest Ethiopia, 2021 (*N* = 406).

Variables	Frequency	Percentage
**Gravidity**		
Primigravida	120	29.56
Multigravida	286	70.44
**ANC**		
Yes	387	95.32
No	19	4.68
**Gestational age**		
Preterm	140	34.48
Term	253	62.32
Post term	13	3.20
**Place of delivery**		
Hospital	333	82.02
Health center	58	14.29
Home	15	3.69
**Mode of delivery**		
SVD	271	66.75
CS	70	17.24
Instrumental	65	16.01
**History of abortion**		
Yes	47	11.58
No	359	88.42
**History of still birth**		
Yes	35	8.62
No	371	91.38
**Cervical cerclage on current pregnancy**		
Yes	1	0.25
No	405	99.75
**ROM**		
Yes	191	47.04
No	215	52.96
**Latency period**		
<18 h	175	91.62
≥18 h	16	8.38
**Induction of labor**		
Yes	68	16.75
No	338	83.25
**Duration of labor (hour)**		
<6	180	44.33
6–12	193	47.54
13–24	21	5.17
>24	12	2.96
**Hypertensive disorder during current pregnancy**		
Yes	65	16.01
No	341	83.99
**Chorioamnionitis**		
Yes	21	5.17
No	385	94.83
**HIV/AIDS**		
Positive	5	1.23
Negative	394	97.04
Unknown	7	1.72

### Time to death of neonates admitted with sepsis

**Figure 1:**
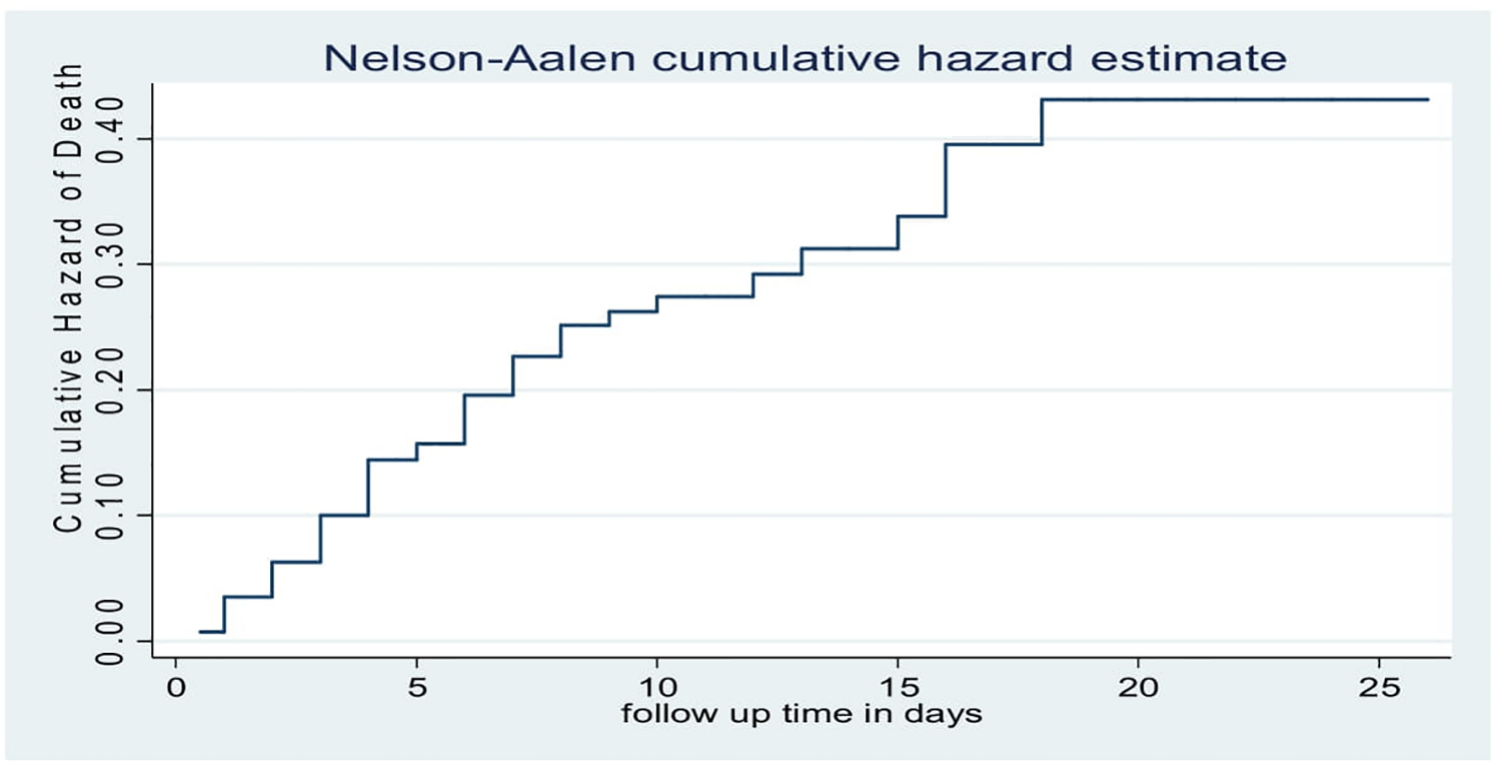
Nelson–Aalen cumulative hazard estimate of neonates admitted with sepsis at N1CU in referral hospitals of northwest Ethiopia, 2021 (*N *= 406).

A total of 406 neonates admitted with sepsis were followed for a median follow-up period of 6 days (IQR: 3–8 days), with minimum and maximum follow-up periods of half a day and 26 days, respectively. Of the total study subjects, 75 (18.47%, 95% CI: 14.82, 22.60) neonates died during the study period. The incidence rate of death was 28 (95% CI, 22, 35) per 1,000 person-days of observation, with a total follow-up time of 2,677 person-days of observation. The cumulative hazard of death among neonates admitted with sepsis on the first, third, sixth, eighth, and 26th day of admission were 3.53% (95% CI: 2.09, 5.86), 9.68% (95% CI: 7.07, 13.19), 18.08% (95% CI: 14.22, 2.283), 22.57% (95% CI: 17.96, 28.15), and 35.51% (95% CI: 26.77, 46.07), respectively (Figure 1).

**Figure 2:**
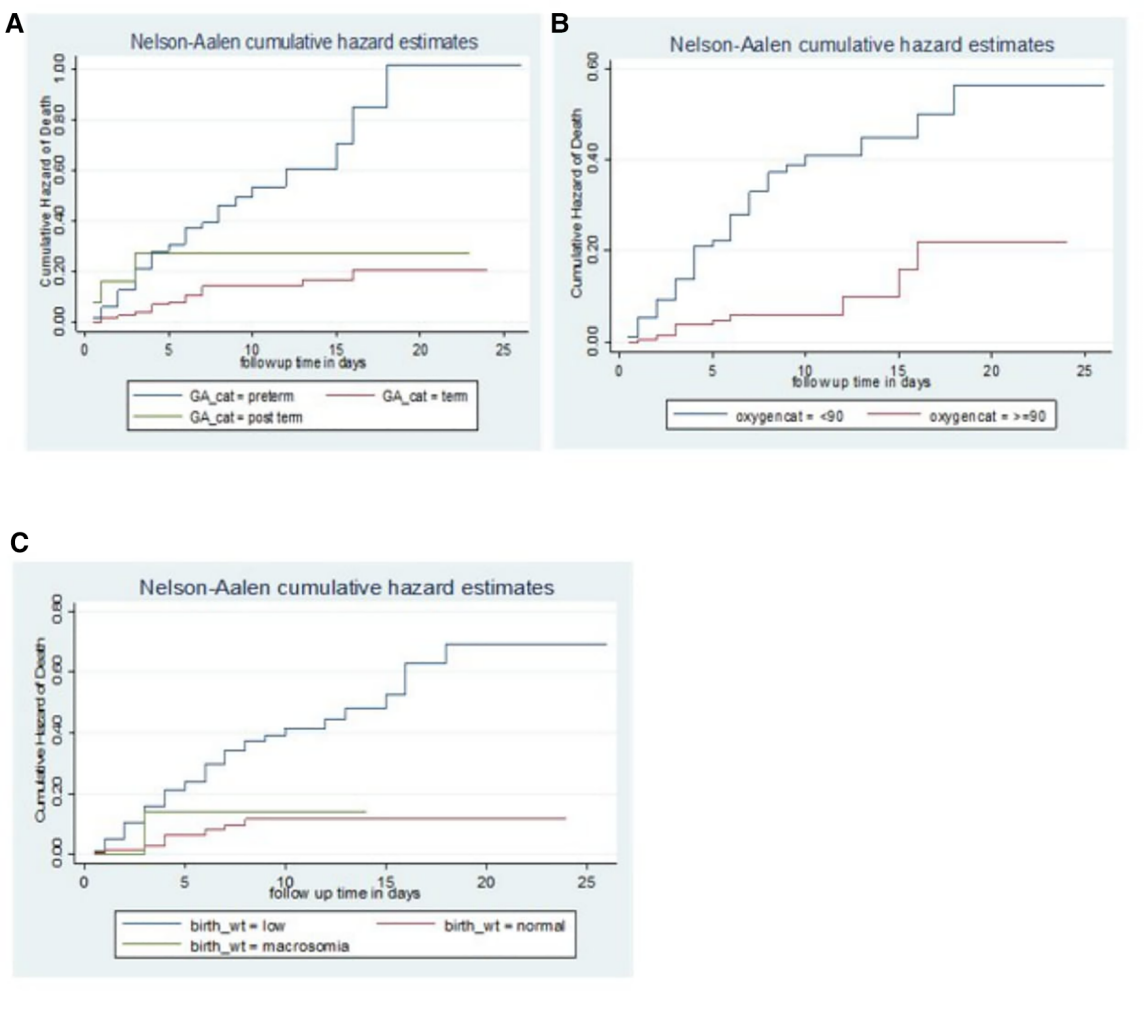
Nelson–Aalen cumulative hazard curve showing different hazards across different categories of the covariates (gestational age (**A**), oxygen saturation (**B**), and birth weight (**C**)).

The Nelson-Aalen curve shows that the hazard of death among neonates admitted with sepsis differs across different baseline clinical characteristics of the neonates. Preterm neonates, low birth weight neonates, and neonates with less than 90% oxygen saturation at admission had a higher hazard of mortality as compared to their counterparts (Figure 2).

### Model comparison and model fitness

Cox PH and three parametric models (Exponential, Weibull, and Gompertz) were fitted to identify the predictors of death among neonates admitted with sepsis. The exponential regression model was selected as the best model with the lowest AIC and BIC. The Cox proportional hazard (PH) assumption was fulfilled, as shown both graphically by the log-minus-log function and statistically by the Schoenfeld residual test (global test, *X*^2^ = 20.80, *P*-value = 0.7035).

### Model fitness

**Figure 3:**
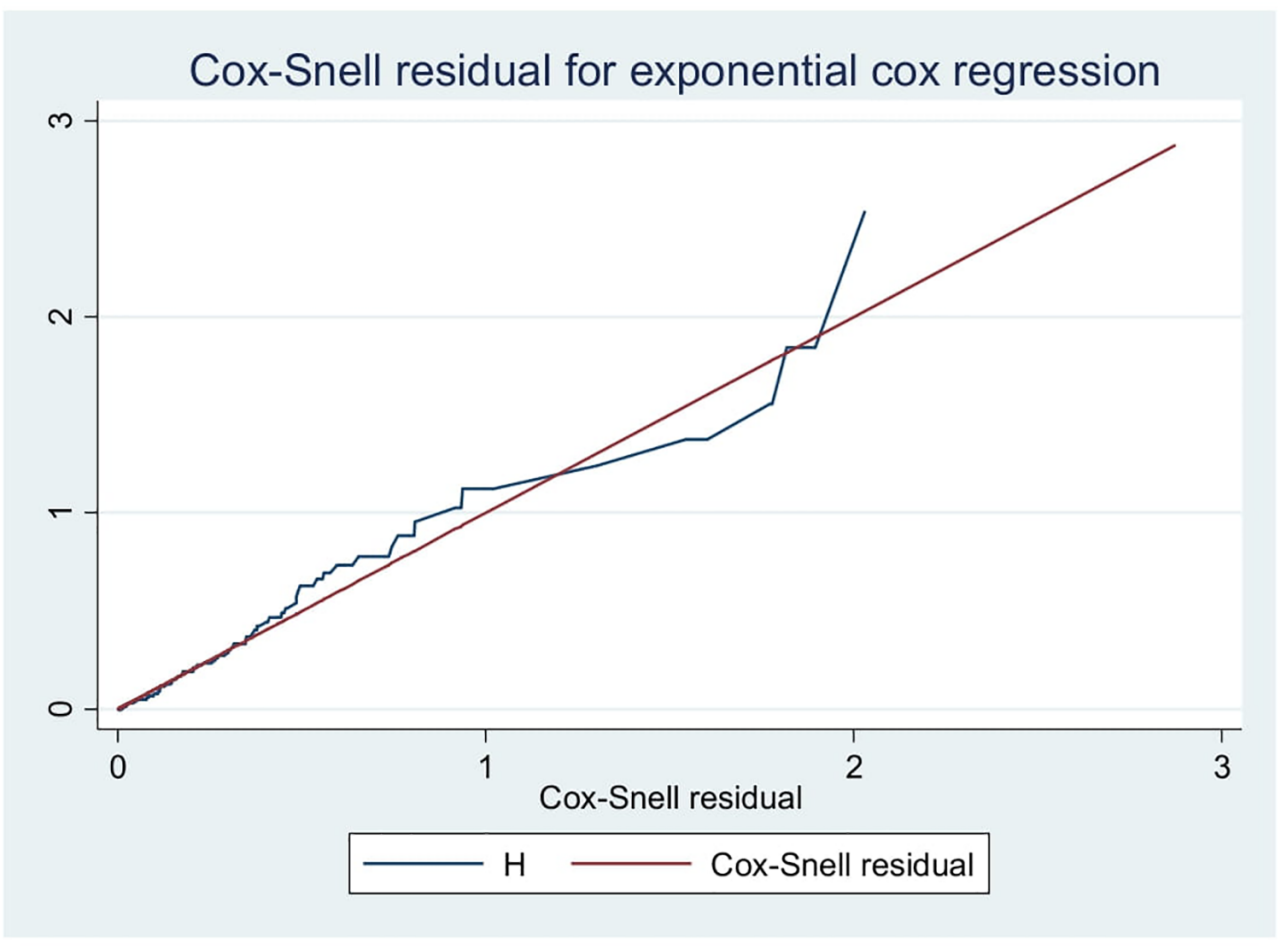
Cox-Snell residual test for overall adequacy of the model fitted for time to death among neonates admitted with a diagnosis of neonatal sepsis.

The overall goodness of fit of the exponential regression model was tested by applying the Cox-Snell residual plots. As shown in Figure 3, the line associated with the Cox-Snell residual of the exponential Cox regression model was closer to 45° straight lines of the origin which indicate that model is well-fitted.

### Predictors of death among neonates admitted with sepsis

On the multivariable exponential regression; duration of labor, breastfeeding status of the neonate, gestational age, birth weight, baseline oxygen saturation, and experiencing RDS at admission were significant predictors of death among neonates admitted with sepsis.

The hazard of neonatal death was two times (AHR = 2.06, 95% CI: 1.02, 4.15) higher among preterm neonates as compared to term neonates. Neonates who were delivered with greater than 24 h of labor duration had four times (AHR = 3.89, 95% CI: 1.38, 11.01) higher risk of death than those who were delivered with 6–12 h of labor duration. The hazard of neonatal death was 2 times (AHR = 2.12, 95% CI: 1.01, 4.43) higher for neonates whose weight was less than 2,500 g compared to normal birth weight neonates. The hazard of death among breastfed neonates was reduced by 57% (AHR = 0.43, 95% CI: 0.23, 0.80) as compared to neonates who were not breastfed. The hazard of neonatal death was two times (AHR = 1.77, 95% CI: 1.02, 306) higher among neonates with respiratory distress syndrome as compared to their counterparts. Neonates whose oxygen saturation was less than 90% at admission had two times (AHR = 2.23, 95% CI: 1.07, 4.61) higher hazard of death than neonates with oxygen saturation ≥90% (Table 4).

**Table 4:** Predictors of death among neonates admitted with neonatal sepsis at NICU of northwest Ethiopia referral hospitals, 2021.

Variables	Outcome		CHR (95% CI)	AHR (95% CI)	*P*-value
	Death	censored			
**Age**					
18–24 years	18	48	1.91 (1.10,3.31)*	1.98 (0.99, 3.94)	0.053
25–34 years	42	234	1	1	
≥35 years	15	49	1.70 (0.94,3.06)	1.24 (0.63, 2.43)	0.53
**Occupation**					
Civil servant	5	58	0.41 (0.16, 1.04)	0.97 (0.36, 2.58)	0.95
Merchant	24	104	0.96 (0.58, 1.58)	1.54 (0.86, 2.74)	0.15
NGO worker	4	13	1.53 (0.55, 4,27)	1.25 (0.41, 3.79)	0.69
Housewife	42	156	1	1	
**Family size**					
<5	41	222	1	1	
≥5	34	109	1.41 (0.89, 2.22)	1.17 (0.67, 2,02)	0.58
**Gestational age**					
Preterm	47	93	3.95 (2.43, 6.41)**	**2.06 (1.02, 4.15)**	0.04
Term	25	228	1	1	
Post term	3	10	2.49 (0.75, 8.26)	2.40 (0.56, 10.26)	0.24
**Place of delivery**					
Hospital	58	273	1	1	
Health center	11	47	0.97 (0.51,1.85)	0.67 (0.32, 1.40)	0.29
Home	6	11	2.09 (0.90, 4.84)	2.61 (0.95, 7.15)	0.06
**Mode of delivery**					
SVD	57	214	1		
CS	10	60	0.68 (0.35, 1.33)	1.18 (0.48, 2.89)	0.73
Instrumental	8	57	0.48 (0.23, 1.01)	1.18 (0.47, 2.95)	0.73
**Duration of labor**					
<6 h	34	146	1.17 (0.72, 1.89)	0.82 (0.46, 1.47)	0.51
6–12 h	32	161	1	1	
13–24	3	18	0.79 (0.24, 2.59)	0.98 (0.28, 3.42)	0.96
>24	6	6	3.87 (1.62, 9.25)*	**3.89 (1.38, 11.01)**	0.01
**Chorioamnionitis**					
Yes	7	14	2.14 (0.98, 4.65)	1.64 (0.62,4.35)	0.32
No	68	317	1	1	
**Birth weight**					
Low	61	156	3.89 (2.14, 7.08)**	**2.12 (1.01,4.43)**	0.05
Normal	13	165	1	1	
Macrosomia	1	10	1.68 (0.22, 12.83)	1.77 (0.21,14.96)	0.60
**Breastfeed**					
Yes	16	214	0.19 (0.11, 0.32)**	**0.43 (0.23, 0.80)**	0.008
No	59	117	1	1	
**RDS**					
Yes	33	60	2.69 (1.70,4.24)**	**1.77 (1.02, 306)**	0.041
No	42	271	1	1	
**Dehydration**					
Yes	19	29	2.85 (1.69, 4.79)**	1.24 (0.67, 2.31)	0.49
No	56	302	1	1	
**O_2_ saturation**					
<90%	65	181	4.25 (2.18,8.27)**	**2.23 (1.07, 4.61)**	0.03
≥90%	10	150	1	1	
**Hypoglycemia**					
Yes	22	41	2.71 (1.65, 4.45)**	1.66 (0.91, 3.03)	0.098
No	53	290	1	1	
**Temperature**					
<35.5°C	46	151	1.77 (1.00, 3.12)	1.71 (0.91, 3.20)	0.09
35.5°C–37.5°C	16	97	1	1	
>37.5°C	13	83	0.97 (0.46, 2.01)	1.91 (0.83, 4.43)	0.13

Bold values indicates statistically significant variables. *Statistically significant at P-value 0.05. **Statistically significant at P-value 0.001.

## Discussion

The findings of multivariable exponential regression analysis of this study identified; gestational age less than 37 weeks (prematurity), duration of labor >24 h, birth weight <2.5 kg, not breastfeeding, respiratory distress syndrome, and Oxygen saturation <90% at admission as predictors of mortality among neonates admitted with sepsis. In this study, 18.47% (95% CI 14.82, 22.60) of neonates died during the follow-up period. The incidence rate of death was 28 (95% CI, 22, 35) per 1,000 person-days of observation, with a total follow-up time of 2,677 person-days of observation. This finding is in line with studies conducted in southwest Ethiopia, in which the death rate associated with sepsis was 14.7% (14), Jimma (22.4%) (18), and Turkey 22.2% (19).

This finding is lower than the study conducted in central India where the mortality rate was 38.24% (20). This inconsistency might be related to the source of data that the Indian study used only referred neonates with maternal and neonatal risk factors of sepsis. Neonates with maternal and neonatal risk factors may have a higher chance of death. Our study considered neonates admitted to NICU regardless of maternal and fetal status. The rate was lower than other studies conducted in Iraq (44.2%) (11), Nigeria (68%) (10), and Brazil (26.1%) (21).

The reason for variation may be due to the current government's attention to reduce neonatal mortality through sustainable development goals.

On the other hand, the rate of neonatal death due to sepsis is higher than studies conducted in North West Ethiopia (4%) (22), western India (7%) (23), Mexico (9.5%) (24), and Malaysia (3.3%) (25). This difference might be explained by socio-economic and health service-related factors.

The current study revealed that low birth weight is a predictor of neonatal mortality among neonates admitted with sepsis. The hazard of mortality doubles for low birth weight neonates when compare to normal birth weight neonates. This finding is in line with studies conducted in Turkey (19), Mexico (24), Pakistan (1), and Nigeria (26). It is known that low-birth weight babies often have difficulty staying warm in normal temperatures and an inability to maintain their body temperature because they have so little body fat. This may increase the risk of death.

Prematurity had shown a significant association with the risk of sepsis-related neonatal mortality with the hazard of death 2 times higher among neonates born before 37 completed weeks of gestation compared to term neonates. This result is consistent with studies conducted in, Durame southern Ethiopia (27), Pakistan (1), Mexico (24), and India (23). It is well known that premature neonates face numerous physiologic challenges and fatal conditions as they adjust to extrauterine life and may not respond to treatments.

The current study showed that neonates who breastfeed after delivery had a reduced risk of death by 57% in comparison with their counters. A study conducted in India also revealed feeding as a protective factor against mortality in babies with neonatal septicemia (28) and this result is supported in previous studies done in Ethiopia (17) and Thailand (29). Since breast milk is a source of nutrients and bioactive factors that help to optimal growth and development, and to fight infections, the risk of death might be reduced in this group of neonates. It is also good to note that neonates who are severely sick might not be able to suck breast milk (30).

In this study, respiratory distress syndrome was identified as the predictor of neonatal mortality from sepsis. Neonates with respiratory distress syndrome were two times more likely to die of neonatal sepsis. This finding is comparable with previous studies conducted in Ethiopia (22, 27) and Nigeria (31). This could be because babies with respiratory distress don’t have surfactant that helps to prevent the collapse of small air sacs in the lungs which increases the risk of neonatal mortality.

This study shows that neonates who were delivered with greater than 24 h of labor duration had four times higher risk of death than those who were delivered with 6–12 h of labor duration. This result is supported by previous studies done in central and southern Ethiopia (32, 33). The possible reason is that prolonged labor might lead to obstructed labor which frequently results in asphyxia that can result in stillbirth, neonatal demise, cerebral palsy, and developmental disabilities (34).

In this study, neonates who had oxygen saturation less than 90% had two times higher hazard of death than neonates with oxygen saturation ≥90%. This finding is supported by other studies done on preterm infants (35, 36). This can be explained by the fact that low oxygen saturation in the blood can affect the oxygen concentration in the body's tissues, including the organs and muscles which results in cell death (37).

## Limitations of the study

The gold standard for the diagnosis of sepsis is culture. However, this study identifies sepsis cases based on clinical manifestation. This may have an impact on the incidence of neonatal mortality among neonates with sepsis, because neonates with sepsis may be negative for their cultures.

## Conclusion

The incidence of neonatal mortality among neonates admitted with the diagnosis of neonatal sepsis was high. Gestational age less than 37 weeks (prematurity), duration of labor >24 h, birth weight <2.5 kg, not breastfeeding, respiratory distress syndrome, and oxygen saturation <90% were significant predictors of neonatal mortality. Therefore, early detection and appropriate management of patients’ presentations like respiratory distress syndrome and low oxygen saturation are necessary to reduce neonatal sepsis-related mortality. Special attention should be given to low birth weight and premature neonates and mothers should be encouraged to breastfeed their newborns after delivery.

## Data Availability

The original contributions presented in the study are included in the article/Supplementary Material, further inquiries can be directed to the corresponding author.
